# Role of Impulsivity and Emotion Dysregulation Dimensions on Core Characteristics of Binge Drinking among University Students

**DOI:** 10.5334/pb.1167

**Published:** 2022-12-22

**Authors:** Farid Benzerouk, Zoubir Djerada, Mickaël Naassila, Sarah Barrière, Arthur Kaladjian, Fabien Gierski

**Affiliations:** 1 Department of Psychiatry, Marne Public Mental Health Institution & Reims University Hospital, 51100 Reims, France; 2 University of Reims Champagne Ardenne, Cognition Health Society Laboratory (C2S – EA 6291), Reims, France; 3 Department of Pharmacology, CHU Reims, 51100 Reims, France; 4 Research Group on Alcohol and Dependences, INSERM & University of Picardy Jules Verne, Amiens, France

**Keywords:** Alcohol, Binge Drinking, positive urgency, negative urgency, (lack of) premeditation, sensation seeking, DERS impulse

## Abstract

Binge drinking refers to a pattern of alcohol consumption that leads to rapid intoxication followed by withdrawal and abstinence periods. This study aimed to investigate the potential differential contributions of impulsivity and emotion regulation difficulties to core characteristics of binge drinking (consumption speed, frequency of binge drinking episodes, and the ratio of binge drinking episodes) among a sample of non-abstainers college students. One thousand and five hundred fifty-five participants (17–25 years old) completed the UPPS-P Impulsive behavior scale, the Difficulties in Emotion Regulation Scale (DERS) and measures related to alcohol consumption patterns and affects by means of an online survey. Multiple regression analyses showed that UPPS-P sensation seeking, lack of premeditation, positive, and negative urgency dimensions were significantly associated with binge drinking core characteristics. More specifically, lack of premeditation, and sensation seeking dimensions were associated with speed of drinking, frequency of binge drinking epiosodes, and the ratio of binge drinking episodes. Positive urgency was associated with speed of drinking, and the ratio of binge drinking episodes while negative urgency was negatively associated with speed of drinking. DERS impulse dimension was associated with speed of drinking, DERS awareness dimension was negatively associated with the frequency of binge drinking episodes, and DERS goals dimension was significantly associated with the ratio of binge drinking episodes. Furthermore, patterns of drinking were independently associated with sex, depression and anxiety scores. These findings may help to plan and develop interventions aimed at addressing binge drinking in young adults by targeting impulsivity and emotion dysregulation.

## Introduction

Alcohol consumption is the leading risk factor for premature mortality and disability among individuals aged 15 to 49 years old, accounting for 10% of all deaths in this age group (WHO, 2020). Moreover, a binge pattern of alcohol consumption (binge drinking (BD)) is highly prevalent in this population (WHO, 2020). According to the National Institute on Alcoholism and Alcohol Abuse (NIAAA), BD is “a pattern of drinking that brings blood alcohol concentration (BAC) levels to 0.08 g/dL, levels which typically occur after 4 drinks for women and 5 drinks for men—in about 2 hours” (NIAAA, 2011). Considering this definition, BD refers to a pattern of consumption that leads to rapid intoxication (Rolland & Naassila, 2017). Moreover, it is associated in an alternative way with withdrawal symptoms and abstinence periods (Maurage et al., 2020). Among young adults, BD has been related to a large set of harmful consequences, including injuries, brain and cardiovascular damages, and alcohol disorders (Jones et al., 2018; Kuntsche et al., 2017; Piano et al., 2017). BD has also been associated with cognitive and emotional impairments (Lannoy et al., 2021; Lees et al., 2019; Stephens & Duka, 2008). Moreover, continued BD pattern during emerging adulthood is suggested to lead to gray matter abnormalities in regions involved in reward processing, emotional regulation and executive functions (Pérez-Garcia et al., 2022). BD is also associated with a reduced quality of life (Dormal et al., 2018).

Given this Public Health concern, studies aimed to identify factors associated with BD. Several of them, as described below, highlighted the important role of impulsivity and others pointed to emotional regulation difficulties among binge drinkers.

Impulsivity is viewed as a multi-faceted construct consisting of several related domains. One of these domains includes the so-called “impulsive personality traits” representing dispositional tendencies toward impulsive behavior. It is typically measured using self-report questionnaires such as the UPPS-P Impulsive Behavior Scale (Cyders et al., 2007; Whiteside & Lynam, 2001a). This model posits that impulsivity is composed of five different dimensions named negative urgency (NU), positive urgency (PU), (lack of) premeditation (PREM), (lack of) perseverance (PERSE), and sensation seeking (SS). Using this model, Bø and collaborators (2016) showed that the intensity of BD was associated with NU, suggesting that the BD pattern was displayed in reaction to negative emotional states, and might be conceptualized as a maladaptive and short-term emotional coping. However, Tran et al. (2018) found that both urgency dimensions were of importance in emerging adults’ problematic alcohol use who may use alcohol to avoid negative mood states and further enhance positive mood states. Moreover, Adan et al. (2017) found that high impulsivity (i.e. poor planning skills, difficulty in maintaining attention, and risk-taking behavior) and high SS were related to BD episodes. Thus, the scores of impulsivity and SS are related to the number of drinks per episode, but also to the frequency of BD (see for review: Adan et al. (2017)). Both impulsivity and SS traits, when present together, have been suggested to contribute to a ‘disinhibited personality’ (Castellanos-Ryan et al., 2011). Taking these results into consideration, Maurage et al. (2019) also proposed a comprehensive overview of the involvement of impulsivity in BD, arguing that current BD habits are mostly determined by high SS and strong PREM and that alcohol-use problems rely on intense NU, PU and PERSE (Maurage et al., 2019).

Rather than being related merely to impulsivity, BD has been associated with emotional processes and, more specifically, with emotion regulation difficulties. According to Gross (Gross, 2015), emotion regulation refers to “shaping which emotion one has, when one has them, and how one experiences or expresses these emotions”. Emotion regulation is conceptualized as involving emotion regulation abilities (i.e. the awareness and understanding of emotions, the acceptance of emotions, the ability to control impulsive behaviors and behave in accordance with desired goals when experiencing negative emotions), and emotion regulation strategies (i.e. use situationally appropriate emotion regulation strategies flexibly to modulate emotional responses as desired in order to meet individual goals and situational demands). Strategies can include adaptive ones such as reappraisal, problem-solving, acceptance, and maladaptive ones such as avoidance, rumination, and suppression. The relative absence of any or all of these abilities and strategies would indicate the presence of difficulties in emotion regulation or an emotion dysregulation (Gratz & Roemer, 2004).

Studies on emotion regulation in BD are recent and very few. Results among young adults showed that higher negative intensity (i.e. temperamental responsivity to emotions) predicts greater coping-motivated drinking, and that this relationship is partially mediated by problems with emotional clarity and limited regulation strategies (Veilleux et al., 2014). Aurora and Klanecky (2016) found that students with high emotion regulation difficulties engage in more severe alcohol use behaviors in an effort to alter their emotional experiences. Poncin et al. (2017) found that emotional distress was related to more self-blame, rumination, and maladaptive regulation strategies in binge drinkers students. These authors also found that this group also exhibited sensitivity to self-stressors with difficulties of emotion regulation. However, according to Lannoy et al. (2021), impaired emotion regulation in BD is not a robust affirmation. Nevertheless, beyond the overall BD phenotype, BD is a complex behavior that is characterized as much by the speed of consumption as by the frequency of intoxication or the percentage of drunkenness episodes (i.e., ratio of drunkenness episodes to the total number of drinking episodes) (Townshend & Duka, 2002). However, data on emotion regulation and on its impact on BD core characteristics are still sparse.

An improved knowledge on BD core characteristics is a necessary step to develop better-targeted prevention and intervention programs in BD. For instance, in a part of an alcohol pharmacotherapy trial of naltrexone, Bold et al. (2017) found that, in young adults (18–25 years-old), both urgency dimensions (i.e. negative and positive) significantly moderated relations between daily affect and intoxication and that young adults who had a tendency to be more reactive to heightened emotional states were more likely to drink to intoxication on days of elevated positive or negative affects, compared to those with lower urgency traits (Bold et al., 2017). In this context, it should be of interest to better characterise facets of impulsivity and emotion regulation related to BD core characteristics.

The aim of the present study was to examine the contribution of impulsivity and emotion regulation difficulties to binge drinking observed in young adults. More specifically, we sought to individuate factors that were significantly related to the core components of this drinking pattern (i.e. speed of drinking, number of times being drunk in the previous 6 months and the percentage of times getting drunk when drinking).

## Material and Methods

### Participants and procedure

We conducted an internet survey-based study on alcohol use among students from several universities located in eastern France, which consisted primarily of Caucasians. The survey was completed anonymously. The recruitment information e-mail outlined the purpose of the study and reminded participants that they were under no obligation to participate. All participants provided online informed consent before starting. No compensation was given.

A total of 1,658 College students, aged between 17 years old and 25 years old, completed the whole survey. Based on the Alcohol Use Disorders Identification Test (AUDIT; Saunders et al., 1993) we filtered-out abstainers (AUDIT–1 = 0), individuals with a daily or almost daily consumption of six or more drinks (AUDIT–3 = 4) and individuals who declared having needed a first alcohol consumption in the morning to get themselves going after a heavy drinking session (AUDIT–6 > 0). In line with Maurage et al. (2020), this procedure was conducted in order to exclude participants with a pattern of alcohol use disorder. The final sample consisted of 1,555 participants (981 women and 574 men), with a mean age of 20.09 years old (S.D. = 1.86; range = 17–25). The characteristics of the final sample are displayed in Table 1.

**Table 1:** Socio-demographic and clinical variables of the sample (N = 1,555).


	MEAN	SD	RANGE

Age	20.09	1.86	17–25

Binge Drinking score	18.34	15.65	3.33–134

Consumption speed	2.15	1.37	0.33–7.00

Drunkenness episodes (6-month)	4.41	8.68	0–100

Drunkenness ratio (6-month)	26.77	23.92	10–100

AUDIT Total score	6.42	4.65	1–28

Beck Depression Inventory	6.12	4.91	1–31

STAI-Trait	44.31	10.80	21–79

Negative urgency	9.04	2.88	4–16

Positive urgency	10.34	2.59	4–16

Premeditation (lack of)	7.35	2.35	4–16

Perseverance (lack of)	7.33	2.46	4–16

Sensation Seeking	9.79	3.03	4–16

DERS Non-Acceptance	12.59	5.64	6–30

DERS Goals	14.60	5.12	5–25

DERS Impulse	11.64	4.93	6–30

DERS Awarness	16.32	4.43	6–30

DERS Strategies	18.10	6.95	8–40

DERS Clarity	10.81	3.91	5–25

DERS Total score	84.06	20.97	36–163


*Note*: Data are presented as Mean (SD); BD: binge drinking; AUDIT: Alcohol Use Disorders Identification Test; STAI: State-Trait Anxiety Inventory; DERS: Difficulty in Emotion Regulation Scale.

### Measures

#### Alcohol use

A revised version of the Alcohol Use Questionnaire (AUQ-R; Townshend & Duka, 2002) was used to calculate a binge drinking score. This score was calculated for all participants on the basis of the information provided regarding core characteristics of BD: consumption speed (average drinks per hour), drunkenness episodes (number of times being drunk in the previous 6 months) and the drunkenness ratio (percentage of times getting drunk when drinking). The AUQ-R binge score is a validated and widely used method for exploring binge drinking (e.g., Czapla et al., 2015; Rae et al., 2020; Townshend & Duka, 2005; Townshend et al., 2014).

The Alcohol Use Disorders Identification Test (AUDIT; Saunders et al., 1993) was developed as a screening tool by the World Health Organisation (WHO) for the early identification of problem drinkers. We used the French version of this scale, which consists of 10 questions regarding recent alcohol use, alcohol dependence symptoms, and alcohol-related problems. The summary score ranges from 0, indicating no presence of problem drinking behavior, to 40, indicating marked levels of problem drinking behavior and alcohol dependence. The Cronbach’s alpha for the full sample on this scale was .774.

#### Impulsivity and Emotion dysregulation

The UPPS-P Impulsive Behavior Scale (Billieux et al., 2012) consists of 20 items that evaluate different facets of impulsivity labeled negative urgency (NU; 4 items), positive urgency (PU; 4 items), (lack of) premeditation (PREM; 4 items), (lack of) perseverance (PERSE; 4 items), and sensation seeking (SS; 4 items). NU measures the tendency to act impulsively due to negative affect. PU measures the tendency to act impulsively due to positive affect. PREM refers to the tendency to act rashly without first reflecting upon the decision to act. PERSE reflects a tendency not to complete projects. SS involves motivation to experience novelty. Cronbach’s alpha in our sample were 0.785 for NU, 0.741 for PU, 0.818 for PREM, 0.870 for PERSE, and 0.848 for SS.

The Difficulty in Emotion Regulation Scale (DERS; Gratz & Roemer, 2004) is a 36-item self-report questionnaire used to assess difficulties with emotion regulation. The DERS assesses six different aspects of emotional regulation, including non-acceptance of emotional responses (Non-Acceptance), difficulty engaging in goal-directed behavior (Goals), impulse control difficulties (Impulse), lack of emotional awareness (Awareness), limited access to emotional regulation strategies (Strategies), and lack of emotional clarity (Clarity). The Non-Acceptance subscale evaluates one’s feelings about emotional responses, with higher scores reflecting negative feelings about emotional responses. The Goals subscale assesses the ability to accomplish goals in the midst of emotional states, with higher scores indicating greater difficulty accomplishing and concentrating on tasks when one is experiencing negative emotions. The Impulse subscale measures one’s ability to regulate behaviour while under emotional distress, with higher scores indicating more impaired regulation. The Awareness subscale assesses the ability to attend to and acknowledge the significance of emotions, with higher scores reflecting lower awareness. The Strategies subscale evaluates one’s ability to influence emotional states, with higher scores indicating lower capacity to change how one feels (evaluation of their ability to influence their emotional states). The Clarity subscale measures the extent to which individuals understand the emotions they are feeling, with higher scores indicating a poorer understanding of feelings. The DERS has demonstrated good internal consistency, construct and predictive validity, and test-retest reliability. We used the validated French version (Dan-Glauser & Scherer, 2012). The DERS total score demonstrated a Cronbach’s alpha of 0.916 in the present study. It was 0.877, 0.879, 0.778, 0.724, 0.874, and 0.787 respectively for the six different subscales described above.

#### Other assessments (depression severity and trait anxiety level)

Depression symptoms severity was assessed with the shortened Beck depression inventory (BDI; Beck et al., 1996). This is a widely used self-report scale consisting of 13 items, which has been validated in French (Collet & Cottraux, 1986). The total score is obtained by adding the scores of the 13 items and ranges from 0 to 39, higher scores indicating greater depression symptoms. The Cronbach’s alpha for the current study was 0.834.

Anxiety symptoms severity was assessed with the Spielberger State–Trait Inventory (STAI; Spielberger & Gorsuch, 1983), which is a 40-item scale, using a 4-point Likert scale for each item. In our study, this scale was used to measure trait anxiety (how dispositionally anxious a person is across time and situations). The Cronbach’s alpha for the current study was 0.913.

#### Statistical Analysis

Means and standard deviations (SD) for continuous measurements were reported when appropriated. Cronbach’s alpha was calculated for each scale and subscale.

The association between demographic, clinical variables and BD core characteristics (i.e. consumption speed, drunkenness episodes, drunkenness ratio) in the whole sample was assessed by Pearson’s correlation and by generalized linear regression analyses using a forward variables selection. All variables were considered clinically relevant and selected for all regression models. BD core characteristics were included as the dependent variables, all impulsivity and emotion dysregulation dimensions as the independent variables in step 2 while controlling the other covariates in the first step (sex, age, depression and anxiety symptoms severity) for confounding effects. Standardized β coefficients were estimated for all significant associations.

The assumptions of multiple linear regression have been verified (i.e. homogeneity of the variance of the residuals and normality of the residuals). In order to determine if multicollinearity existed in each of our three models, variable inflation factors (VIFs) were calculated for each of the independent variables (from 1.02 to 2.54 with speed of drinking as dependant variable, from 1.01 to 2.2 with drunkenness episodes as dependent variable, from 1.02 to 2.57 with BD ratio as dependent variable). As all of the VIFs are less than 3 (O’Brien, 2007), it was determined that multicollinearity was not an issue.

Statistical analyses were performed using SPSS statistical software version 26.0 (IBM Corp.) and *p*-values < 0.05 were regarded as statistically significant.

## Results

### Socio-demographic and clinical variables

Table 1 summarizes the characteristics of the final sample.

### Bivariate correlations between core characteristics of BD, clinical characteristics, impulsivity and emotion dysregulation dimensions scores (Table 2)

**Table 2:** Bivariate correlations made in the whole sample (N = 1,555) between core characteristics of BD, clinical characteristics, impulsivity and emotion dysregulation dimensions scores.


	*1*	*2*	*3*	*4*	*5*	*6*	*7*	*8*	*9*	*10*	*11*	*12*	*13*	*14*	*15*	*16*	*17*

***1 Speed^1^***																	

***2 Drunkenness^2^***	.42**																

***3 Drunkenness ratio***	.51**	.61**															

***4 Sex***	.34**	.21**	.15**														

***5 Age***	–.07**	.01	–.004	–.02													

***6 BDI^3^***	.05	–.04	.03	–.11**	–.09**												

***7 STAI^4^***	–.03	–.06*	.02	–.21**	–.07**	.73**											

***8 NU^5^***	.02	–.003	.04	–.13**	–.11**	.25**	.38**										

***9 PU^6^***	.16**	.14**	.15**	–.02	–.10**	.14**	.23**	.54**									

***10 PREM^7^***	.14**	.17**	.14**	.006	–.02	.08**	.09**	.24**	.27**								

***11 PERSE^8^***	.07**	.05*	.06*	.02	–.007	.21**	.22**	.14**	.09**	.45**							

***12 SS^9^***	.27**	.24**	.23**	.17*	–.06*	.03	.02	.16**	.37**	.14**	–.02						

***13 NA^10^***	.003	–.03	.04	–.09**	–.09**	.36**	.46**	.30**	.26**	.03	.11**	.12**					

***14 GOALS***	.03	.02	.08**	–.10**	–.10**	.38**	.49**	.34**	.28**	.13**	.19**	.07**	.43**				

***15 IMP^11^***	.08**	.01	.08**	–.10**	–.10**	.39**	.50**	.47**	.33**	.16**	.10**	.14**	.46**	.52**			

***16 AWAR^12^***	.06*	–.02	.02	.02	.001	.09**	.05	.06*	–.008	.19**	.10**	.05	–.11**	–.12**	–.01		

***17 STRAT^13^***	.01	–.03	.03	–.12**	–.11**	.53**	.66*	.37**	.29**	.07**	.16**	.05*	.58**	.65**	.64**	–.09**	

***18 CLAR^14^***	.06*	.02	.06*	–.04	–.13**	.47**	.55**	.30**	.22**	.16**	.19**	.13**	.40**	.29**	.39**	.28**	.45**


*Note*: ^1^ Speed = Consumption speed, ^2^ Drunkenness = drunkenness episodes in the past 6 months, ^3^ BDI = Beck Depression Inventory, ^4^ STAI = State-Trait Anxiety Inventory (Trait), ^5^ NU = Negative urgency, ^6^ PU = Positive urgency, ^7^ PREM = Premeditation (lack of), ^8^ PERSE = Perseverence (lack of), ^9^ SS = Sensation seeking, ^10^ NA = DERS Non-acceptance, ^11^ IMP = DERS Impulse, ^12^AWAR = DERS Awareness, ^13^STRAT = DERS Strategies, ^14^ CLAR = DERS Clarity. * p < 0.05; ** p < 0.01.

Sex, positive urgency, lack of premeditation, lack of perseverance, sensation seeking, DERS impulse, DERS awareness, and DERS clarity scores were positively correlated with consumption speed. Age was negatively correlated with consumption speed.

Sex, positive urgency, lack of premeditation, lack of perseverance, and sensation seeking scores were positively correlated with BD ratio. Anxiety trait was negatively correlated with the number of drunkenness episodes.

Sex, positive urgency, lack of premeditation, lack of perseverance, sensation seeking, DERS goals, DERS impulse, and DERS clarity scores were positively correlated with BD ratio.

Depression severity score, anxiety score, negative urgency, DERS non-acceptance, DERS Goals, DERS impulse, and DERS strategies scores were negatively correlated with sex. Sensation seeking score was positively correlated with sex.

Depression severity score, anxiety score, negative urgency, positive urgency, senstation seeking, DERS non-acceptance, DERS Goals, DERS impulse, DERS strategies, and DERS clarity scores were negatively correlated with age.

Depression severity score, DERS non-acceptance, Goals, and Impulse scores were highly correlated with DERS strategies score. Positive urgency score was highly correlated with negative urgency score. All other correlations are described in Table 2.

### Associations between BD core characteristics, socio-demographic and clinical variables in the whole sample

Multiple regression analyses (Table 3) showed that sex was significantly associated with the three components of BD, with male displaying higher values. Consumption speed was also significantly related to higher BDI, PU, PREM, SS and IMPULSE scores, and to lower STAI-trait and NU scores. Drunkenness episodes (the number of past 6-month drunkenness episodes) was also related to higher PREM and SS scores and to lower Awarness scores. BD ratio (i.e the percentage of times getting drunk when drinking) was related to lower NU scores and higher PU, PREM, SS and Goals scores.

**Table 3:** Multiple linear regression analyses of the association between binge drinking core characteristics, socio-demographic and clinical variables (N = 1,555).


COVARIATE	WHOLE SAMPLE (N = 1,555)		

	MULTIVARIATE ANALYSES STANDARDIZED β (95% CI), *p*		

	CONSUMPTION SPEED	DRUNKENNESS EPISODES	DRUNKENNESS RATIO

	FINAL MODEL ADJ R^2^ = .19; F(9,1545) = 40.89; *P* < .001	FINAL MODEL ADJ R^2^ = .11; F(7,1547) = 27.77; *P* < .001	FINAL MODEL ADJ R^2^ = .083; F(9,1545) = 16.73; *P* < .001

Sex	.303(.257–.350), *p* < .001	.165(.116–.214), *p* <.001	.121(.071–.171), *p* < .001

Age	–.039(–.084;.007), *p* = .096	.027(–.02–.075), *p* =.255	.021(–.027–.069), *p* =.397

Beck Depression Inventory	.103(.038–.169), *p* = .002	–.008(–.077–.061), *p* =.825	.028(–.042–.097), *p* =.437

STAI-Trait	–.087(–.159 ;–.016), *p* =.017	–.032(–.101–.038), *p* =.374	–.016(–.092–.060), *p* =.682

Negative urgency	–.065(–.124;–.007), *p* =.029		–.061(–.121–.000), *p* =.050

Positive urgency	.087(.029–.145), *p* =.003		.082(.021–.144), *p* =.009

Premeditation (lack of)	.098(.051–.145), *p* <.001	.158(.110–.207), *p* <.001	.097(.047–.147), *p* <.001

Perseverance (lack of)			

Sensation Seeking	.173(.123–.222), *p* <.001	.196(.148–.244), *p* <.001	.175(.123–.228), *p* <.001

DERS Non-Acceptance			

DERS Goals			.065(.009–.121), *p* =.023

DERS Impulse	.072(.016–.128), *p* =.012		

DERS Awareness		–.056(–.104;–.008), *p* =.022	

DERS Strategies			

DERS Clarity			


*Note*: adj R^2^ = Adjusted R-square; BD: Binge Drinking; DERS: Difficulty in Emotion Regulation Scale. STAI: Spielberger State–Trait Inventory. Data are presented in regression standardized β coefficients (β) (95% confident interval), and *p*-value (after controlling for sex, age, depression severity score, and anxiety score), only significant standardized β coefficients (β) (95% confident interval) of the forward procedure are presented.

## Discussion

The aim of the present study was to examine the contributions of impulsivity and emotion dysregulation dimensions to binge drinking core characteristics observed in non-abstainers young adults. Our main findings, were as follows: (1) speed of drinking, was associated with sex, depression symptoms severity score, trait anxiety score, most of impulsivity scores (i.e. NU, PU, PREM, SS), and an emotion dysregulation score (i.e. Impulse), (2) the number of times being drunk in the previous 6 months was associated with sex, and with specific dimensions of impulsivity (i.e. PREM, SS), and emotion dysregulation (i.e. Awareness), (3) the percentage of times getting drunk was associated with sex, NU, PU, PREM, SS impulsivity dimensions scores, and Goals emotion dysregulation score. To the best of our knowledge, in addition to confirming previous results regarding impulsivity, our study is the first to assess contributions of emotion dysregulation, in an integrated model including impulsivity, in this population of emerging adults.

Concerning impulsivity we found that SS (i.e. the tendency to seek novel experiences, sensory pleasure and excitement even if situations are dangerous or risky), was positively associated with the speed of drinking, the number of times being drunk in the previous 6 months, and with the percentage of time getting drunk when drinking. Among impulsivity and emotion dysregulation dimensions, this dimension had the greatest influence on core characteristics of binge drinking. It is suggested that among emerging adults, this dimension is a strong predictor of alcohol use (Stamates & Lau-Barraco, 2017). SS might be characterized by a motivational system that exaggerates the impact of reward and undermines the impact of punishment, that is, the predominance of approach behavioural tendencies rather than avoidance (Bechara et al., 2002). Moreover, this dimension could also reflect a high tolerance for punishment from maladaptive behaviors, that is to say that the negative consequences of alcohol drinking may not be sufficient to deter individuals with high scores on this dimension (Berg et al., 2015). Interestingly, this assumption is in line with findings providing evidence about decreased amygdala/Orbito-Frontal Cortex (OFC) connectivity as a mechanism linking SS to alcohol drinking (Crane et al., 2018). In young adults, high SS could also be linked to future alcohol-use problems if the behavior persists.

Lack of premediation, defined as the tendency to act without thinking, forethought, planning and without adequate consideration of potential outcomes, was also positively associated with the three core characteristics of binge drinking in our study. This dimension of impulsivity is viewed as presenting deficits in conscientiousness (Whiteside & Lynam, 2001b). Deep insight in this dimension lead us to consider its relationship with the efficacy of decision-making processes (Bechara & Van Der Linden, 2005). Taken together, the findings showed that the same impulsivity dimensions (i.e., SS, and PREM) were significant positive predictors of BD habits. These results are in line with data reported by Maurage et al (2019), who suggest that “current BD habits are mostly determined by strong lack of premeditation and high sensation seeking” (Maurage et al., 2019). These deficits could lead to decision-making with little regard to past outcomes or forethought for possible future outcomes so that the negative consequences of BD may be underestimated, and alcohol drinking could progressively lead to the development of an alcohol-use disorder.

Positive urgency was associated with speed of drinking, and with percentage of times getting drunk when drinking. This dimension is suggested to predict more alcohol use during positive emotion states (Cyders et al., 2010). Moreover, it may interact with drinking motives to predict problematic levels of alcohol use, especially among individuals high in the motive to drink to enhance an already positive mood (Cyders et al., 2007). Interestingly, lower NU (a dimension wich refers to acting rashly and impulsively when in extreme distress and wich involves impaired inhibitory control (Cyders & Smith, 2008)) was a significant predictor of speed of drinking and BD ratio. This could be due to the young age of our sample for which alcohol-use problems are not yet present, as this impulsivity dimension refers to drinking consequences (Maurage et al., 2019). Moreover, it may also be because the motive to drink to cope with distress is not a significant expectancy in our population. By showing that postive and negative urgency dimensions were associated with different characteristics of alcohol drinking in young aldults, our results go against recent findings suggesting that these dimensions should be viewed as a single construct (Billieux et al., 2021).

Concerning emotion regulation, we found that the Impulse dimension of the DERS was positively associated with the speed of drinking, and then seems to predispose participants to drink rapidly. The difficulties maintaining behavioral control when distressed, assessed by this DERS Impulse control difficulties subscale, describe individuals who have very strong feelings that are hard to control (Gross, 2015). Moreover, this subscale specifically focuses on feeling “out of control” in emotionally distressing situations. Several studies demonstrate that IMPULSE dimension may be related to an altered emotionality and to deficits in prepotent response inhibition (Johnson et al., 2017, 2020). This dimension has been shown to predict alcohol use and also binge eating (Miller & Racine, 2020). Moreover, it may increase rates of alcohol use among those who drink and number of alcohol-related consequences as shown in a sample of college students (Dvorak et al., 2014). It was also directly associated with greater alcohol problems (i.e. social/interpersonal, academic/occupational, risky behavior, impaired control, poor self-care, diminished self-perception, blackout, and physiological dependence) (Messman-Moore & Ward, 2014; Simons et al., 2017). Goals dimension was a significant positive predictor of the percentage of times getting drunk when drinking. This DERS dimension seems to be related to difficulties in shifting attention and disengaging from a preoccupying affective state (Gross, 2015). It is viewed as a dimension describing individuals who are prone to ruminations. In our study, participants who struggle with ruminations may be at risk to drink to drunkenness to deal with an aversive affective state.

Surprisingly, Awareness dimension was negatively associated with the number of drunkenness episodes in the previous 6 months. A good emotional awareness was shown as facilitating better ability to navigate complex social situations and enjoy relationships (Lane & Smith, 2021). As social relationships with peers have been shown to play a fundamental role in high intensity drinking (Merrill et al., 2021), it is conceivable that good emotional awareness is associated with a wider social circle and increased consumption opportunities. We could therefore hypothesize that this emotion regulation ability is a risk factor of drunkenness episodes, particularly if it is combined with high levels of impulsivity.

As expected, we highlighted an association between sex (i.e. males) and BD core characteristics. Sex had the greatest influence. However, this is still a topic of debate. Indeed, binge drinking in females is maybe increasing faster than binge drinking in males, and, as a result, males’ and females’ binge drinking rates are converging. Further convergence may be hard to predict and cannot be attributed entirely to females increased binge drinking (Wilsnack et al., 2018). Depression symptoms severity score was positively associated with speed of drinking. Accordingly, alcohol consumption has been largely described as a way to cope with depressive symptoms (Kuntsche et al., 2017). In the same vein, our results suggest that quick drinking may be a mean to try to cope “rapidly” with depressive symptoms. Nevertheless, our results also showed that trait-anxiety was negatively related to quick drinking, showing that it still raise the question of what drives binge drinking between negative and/or positive emotional states (Lannoy et al., 2021).

Despite the meaningful findings provided by this study, it is not without limitations. A first limitation is the crosssectional design; therefore, caution is needed in inferring causality. A second limitation is based on the assessment of impulsivity, and emotion regulation through self-reports, which are subjects to possible biases. However, the validity of these questionnaires has been well supported in previous studies and our reliability indices were satisfactory. We must also bear in mind the tendency for high drinkers to underestimate consumption and drinking behavior (Townshend & Duka, 2002). Finally, in this internet survey-based study on alcohol use among students, participants were not asked about their psychopathological history, other substance abuse or dependence, and family history of alcoholism.

## Conclusions

In sum, the present study contributes to the literature by highlighting relationships between binge drinking core characteristics, dimensions of impulsivity, and emotional dysregulation in university students. The combination of impulsivity, and emotional dysregulation could predispose young adults to BD. Moreover, we have shown that lack of premeditation and sensation seeking impulsivity dimensions were associated with the three core features of BD. These findings have important implications for understanding risks for behaviors towards rapid and massive alcohol consumptions. Impulsivity, and emotion regulation processes will serve as important targets for prevention and treatment efforts in at-risk populations.
